# Seroprevalence and Incidence of hepatitis E in Blood Donors in Upper Austria

**DOI:** 10.1371/journal.pone.0119576

**Published:** 2015-03-09

**Authors:** Carina Fischer, Martina Hofmann, Martin Danzer, Katja Hofer, Jennifer Kaar, Christian Gabriel

**Affiliations:** Red Cross Transfusion Service for Upper Austria, Linz, Austria; Virginia Polytechnic Institute and State University, UNITED STATES

## Abstract

**Background:**

In recent years various studies showed, that hepatitis E virus (HEV) is a growing public health problem in many developed countries. Therefore, HEV infections might bear a transmission risk by blood transfusions. The clinical relevance still requires further investigations. The aim of this study was to provide an overview of acute HEV infections in Upper Austrian blood donors as well as a risk estimation of this transfusion-related infection.

**Methods and Findings:**

A total of 58,915 blood donors were tested for HEV RNA using a commercial HEV RT-PCR Kit. 7 of these donors (0.01%) were PCR-positive with normal laboratory parameters in absence of clinical signs of hepatitis. Viral load determined by quantitative real-time PCR showed a HEV nucleic acid concentration of 2,217 293,635 IU/ml. At follow-up testing (2–11 weeks after donation) all blood donors had negative HEV RNA results. Additionally, genotyping was performed by amplification and sequencing of the ORF1 or ORF2 region of the HEV genome. All HEV RNA positive donor samples revealed a genotype 3 isolate. For the antibody screening, anti-HEV IgM and IgG were detected by ELISA. Follow up serological testing revealed that no donor was seropositive for HEV IgM or IgG antibodies at time of donation. Moreover, we verified the prevalence of anti-HEV IgG in 1,203 of the HEV RNA negative tested blood donors. Overall 13.55% showed positive results for anti-HEV IgG.

**Conclusions:**

In the presented study, we investigated HEV infections in blood donations of Upper Austria over 1 year. We concluded that 1 out of 8,416 blood donations is HEV RNA positive. Seroprevalence of anti HEV IgG results in an age-related increase of 13.55%. Therefore, based on this data, we recommend HEV-PCR screening to prevent transmission of hepatitis E virus by transfusion.

## Introduction

Hepatitis E Virus (HEV) is a spherical, single-stranded, positive-sense RNA virus without an envelope that belongs to the genus *hepevirus* in the hepeviridae family [1,2]. Until recently, HEV was mainly recognised as an infection in tropical countries with high endemism and as a travel-associated disease with a low mortality rate [3]. Nowadays, in developed countries, more HEV infections are autochthonous than clearly travel-associated [4]. HEV strains are divided into four major genotypes [5]. HEV genotypes 1 and 2 are restricted to humans and transmitted from person to person via the faecal-oral route e.g. contaminated water. These genotypes mainly occur in developing regions and are associated with epidemic and sporadic hepatitis E infections. HEV genotypes 3 and 4 represent a zoonotic disease and were found in several non-human primates, as well as in pigs, cows, wild boars, deers, rabbits and rodents [5–7]. Genotype 3 is common in industrialized countries, especially in Europe, in North America and in Japan, whereas genotype 4 was mainly identified in China. In Europe the most prevalent (sub) genotypes are 3c, 3e, and 3f [8–11]. In general, HEV genotype 3 and 4 infections are less pathogenic than genotype 1 and 2, they are often anicteric and asymptomatic self-limiting infections in an apparently healthy population like blood donors [12,13]. Transfusion-transmitted HEV infections are rarely reported. However, there is a broad variety of clinical courses in patients who received HEV positive blood products [14,15]. By now, some cases of transfusion-transmitted HEV infections are reported in industrialized countries, for example the first reported case in Japan 2004 [16], or cases in the UK since 2006 [14,17], as well as in Germany 2013 [18]. The usually absence of symptoms and commonly viral clearance often leads to unrecognized transfusion-transmitted HEV cases [4,5]. Caution should be taken in risk groups like pregnant women, children, immunocompromised and transplanted recipients, where HEV-infections may take a deleterious path and pose as a rare, but hazardous transfusion-associated disease, with symptoms like jaundice, abdominal pain, hepatomegaly and splenomegaly, elevated transaminases and nausea [1,4,14–17]. In some cases HEV infection can lead to fulminant liver failure and particularly solid organ transplant recipients often develop a chronic HEV infection, which can lead to liver cirrhosis and usually has to be treated with anti-viral therapy [19–21].

HEV cannot be inactivated in blood products and detection by antibody screening of IgM is not safe enough to exclude HEV in blood products [4,15,22]. Vollmer et al reported that in 4 out of 10 HEV RNA-positive samples HEV-Ag was detectable and HEV-specific IgM antibodies were only detectable in 7 out of 10 HEV RNA-positive donors [23]. Consequently, HEV antibody screening cannot reduce risks of HEV infections and therefore nucleic acid testing (NAT) becomes a gold standard to prevent active viral transmission to a growing recipient population with higher risks [23,24]. Data about IgG seroprevalence in different European countries are available and vary widely (Austria 14.3% [25], Sweden 9.3% [26], France 3.2% [27] and Southwest France 52.5% [28], United Kingdom and North Wales 10% [29], Germany 16.8% [30]). With respect to the seroprevalence, HEV is a widely spread infection among the European population and therefore represents an easily avoidable risk for transfusion-transmitted diseases, regardless whether there are frequently occurring transfusion-transmitted HEV infections or unrecognized, asymptomatic infections. To our knowledge, there are no data about the HEV RNA prevalence in Austria so far. In order to determine the current actual distribution of viremic persons by HEV RNA in the Austrian population, especially in blood donors, 58,915 donors from Upper Austria were tested by PCR and subsequently genotyped. Additionally, 1,203 blood donors as a subset of this population were randomly selected and tested for HEV IgG antibodies in order to find the prevalence of persons who have had a HEV infection in their history and moreover to determine possible differences in relation to different geographical regions. The results of this study allow an estimation of the occurrence of autochthonous, asymptomatic HEV infections in Upper Austria in respect to consider the implementation of HEV RNA screening of blood donations.

## Materials and Methods

### Ethics statement

Informed consent is obtained in the donor questionnaire and signed by the donor. All questionnaires are scanned in an automatic reading system and those with a positive tick are released for further testing. The central ethics committee of Upper Austria released a statement in which it indicated that as long as samples are residual material anyway obtained by donation procedures and where these samples are not used for donor release criteria or further therapeutic use, there is no requirement for an ethics committee vote. Anyway, we still have the requirement of informed consent by the donor questionnaire.

### Sample collection

Voluntary blood donations of the Upper Austrian Red Cross Transfusion Service were tested by PCR for the presence of HEV RNA. Between February 2013 and April 2014, samples were tested in pools of 96 donors, representing a total of 58,915 donations. Samples which were tested positive for HEV RNA were subsequently genotyped and tested for anti-HEV IgG and anti-HEV IgM antibodies at time of donation and at a follow-up appointment (2–11 weeks after donation). Further, HEV RNA positive blood donors were asked to complete a questionnaire to find out if they were travelling abroad in the last year and if they noticed symptoms like diarrhoea, nausea, abdominal cramping, jaundice or colour changes in urine and stool. They were also asked whether they were occupationally in contact with animals or if they have recently eaten raw meat, game or offal. Additional, women were asked whether they were pregnant and have had abortion or other problems in pregnancy in the last year. Furthermore, in the period of October 2013 to January 2014, a subset of the original samples were randomly selected and tested for anti-HEV IgG antibodies.

### HEV RNA detection in blood donors

Blood donation samples were pooled by 96 samples on a Freedom EVO Clinical system (Tecan, Männedorf, Switzerland). HEV RNA was extracted from 1 ml EDTA pooled plasma using the MagNA Pure Compact system (Roche Diagnostics, Mannheim, Germany) and the Nucleic Acid Isolation Kit I—Large Volume. Reverse transcription, amplification and detection were performed on the LightCycler 480 system (Roche Diagnostics) using the RealStar HEV-RT PCR Kit (Altona Diagnostics, Hamburg, Germany). 5 μl of an Internal Control (IC) containing in the kit was added at extraction. A positive IC is required for a valid result. RNA was reverse transcribed for 10 min at 50°C. Denaturation was performed at 95°C for 10 min followed by 40 amplification cycles. Amplification constituted a denaturation step at 95°C for 15 sec, an annealing step at 55°C for 45 sec and an elongation step at 72°C for 15 sec. The sensitivity of the assay, calculated by Probit analysis, was validated on the World Health Organisation (WHO) international standard (IS) strain 6329/10 and showed a 95% detection limit of 11,6 IU/ml (CI 7.48–25.09) HEV RNA and a 99% plausibility of 21,0 IU/ml (CI 12.01–58.70) HEV RNA. Positive pools of 96 samples were tested in subpools of 12 samples and consequently those were deconstructed to confirm the individual HEV RNA positive blood donor. A positive blood sample was retested and confirmed using an additional blood sample. HEV virus load of positive samples was calculated from the Ct-values based on a calibration curve of the first WHO IS strain 6329/10.

### HEV genotyping and sequence analysis of the HEV RNA ORF2 region

HEV genotyping was performed by sequencing the ORF2 region of the genome adapted from Baylis et al [31]. HEV RNA was extracted using the MagNA Pure Compact System. Reverse transcription and amplification was performed on a Veriti Thermal Cycler (Life Technologies, Darmstadt, Germany) using the OneStep RT-PCR Kit (Qiagen, Hilden, Germany) and the following primers: HEV_fw_int (5’-GTYATGYTYTGCATACATGGCT-3’) and HEV_rv_int (5’-AGCCGACGAAATYAATTCTGTC-3’) [31]. HEV RNA was reverse transcribed for 30 min at 50°C and a following initial polymerase activation step for 15 min at 95°C. PCR amplification is composed of a denaturation step for 30 sec at 94°C, an annealing step at 48°C for 30 sec and an elongation step at 72°C for 1 min. HEV RNA is amplified with 45 cycles. Final extension was performed for 10 min at 72°C. The amplification products were analyzed by an agarose gel electrophoresis with GelStar as a Gel Loading Solution (Biozym, Hessisch Oldendorf, Germany) and a peqGOLD Low Range HT DNA-Ladder (Peqlab, Erlangen, Germany). If genotyping of the ORF2 was unsuccessful, the ORF1 region was sequenced using the following primers according to Vollmer et al [32,33]: HEV_ORF1_F_in (5′-CTGCCCTGGCGAATGCT-3′) and HEV_ORF1_R_in (5′-AGCAGTATACCAGCGCTGAACATC-3′). A specific PCR product on the gel was finally purified with ExoSAP-IT as described and sequenced using the BigDye Cycle Sequencing Kit (Life Technologies) on a Genetic Analyzer 3130xl (Life Technologies). Sequence analysis and phylogenetic analysis were assessed using the DNAStar Lasergene 8 software and alignment was done by using the BLAST service from NCBI.

### Serological testing

Serum samples were tested for anti-HEV IgG and anti-HEV IgM antibodies by anti-HEV IgG and anti-HEV IgM ELISA kit (Wantai Biological Pharmacy Enterprise Co., Ltd., Beijing, China). The test and the calculation of the results were performed according to the manufacture’s instruction. The absorbance was measured using Tecan Infinite 200 pro (Tecan). Positive blood donors were tested for anti-HEV IgG and anti-HEV IgM antibodies at time of donation and at a follow-up appointment after donation. To determine the occurrence of past HEV infections in Upper Austria randomly selected blood donations were tested for anti-HEV IgG only. Borderline results in this group were retested. The results were finally reported as positive or negative for presence of anti-HEV IgG or anti-HEV IgM antibodies.

### Statistical analysis

Data analysis and collection was performed using MS Excel 2007 and IBM SPSS Version 21 for Windows. Categorical variables were compared by Chi-Square test or Fisher’s exact test. A p-value of <0.05 was considered as statistically significant. Data were represented including descriptive statistics as means, 95% confidence intervals (CI’s), standard deviation (SD) as wells as total and relative frequencies. Age-dependent risk factor for presence of anti-HEV IgG was constituted by the calculation of odds ratios and a correlation was calculated for age and the presence of anti-HEV IgG.

## Results

### Characteristics and clinical markers of HEV infected blood donors

In the period of February 2013 to April 2014, 58,915 blood donations in Upper Austria were screened for the presence of HEV RNA and resulted in 7 (0.01%; incidence 1:8,416) HEV RNA positive blood donations (Table 1). The age of positive donors ranged from 21 to 52 years with a mean of 35 and there was one woman among the 7 HEV RNA positive donors. All 7 positive blood donors were confirmed shortly after the positive result to report the HEV infection to the Ministry of Health. The donors were retested for HEV RNA at a follow-up appointment to exclude a chronic HEV infection. The donors were asked to participate to the follow-up testing after 6 weeks. Unfortunately, the testing actually was performed 2–11 weeks after donation. One week after the donation all blood donors were confirmed as positive for HEV RNA. At follow-up appointment all blood donors were negative for HEV RNA. Testing for anti-HEV IgG and IgM resulted in negative results for both anti-HEV IgG and anti-HEV IgM antibodies at time of donation, which implies that these donors were in the seronegative phase of infection. At the follow-up appointment all donors were anti-HEV IgG positive and 6 of 7 had positive results for anti-HEV IgM. HEV RNA concentration was calculated by a standard curve (r^2^ = 0.9995) and corresponds to a HEV nucleic acid concentration of 2.2 x 10^3^ to 2.9 x 10^5^ IU/ml (mean 11.1 x 10^4^ IU/ml). All of the HEV RNA positive blood donors showed normal results for alanine aminotransferase (ALT) and normal C-reactive protein (CRP) at time of donation. Detailed results are supported in S1 and S2 Tables.

**Table 1:** Characteristics of HEV RNA positive blood donors.

Donor	Age^a^	Sex	HEV RNA [IU/ml]	Anti-HEV [at time of donation]		Anti-HEV [follow-up control]^b^		Follow up appointment [weeks after donation]	HEV genotype
				IgG	IgM	IgG	IgM		
**1**	33	F	2,2 x 10^3^	-	-	+	+	6,1	3f
**2**	44	M	2,9 x 10^5^	-	-	+	+	4,1	3f
**3**	52	M	3,3 x 10^4^	-	-	+	-	11,0	3
**4**	23	M	2,5 x 10^5^	-	-	+	+	5,1	3
**5**	40	M	8,0 x 10^4^	-	-	+	+	7,1	3
**6**	21	M	2,1 x 10^4^	-	-	+	+	2,0	3
**7**	32	M	9,1 x 10^4^	-	-	+	+	5,2	3f

F: female; M: male;-: no antibody; +: positive

^a^Age at time of donation

^b^control at least 2 weeks after donation

Analysing the questionnaires, there is no commonality between the HEV positive blood donors. 3 of 7 donors stated that they had diarrhoea or colour changes in stool in the last 6 months, which unfortunately are unspecific symptoms especially during a period of 6 months. Actually they didn´t present any symptoms and didn´t have health problems at time of infection, respectively. One of the 7 positive blood donors is occupationally in contact with animals. 5 of 7 blood donors had a trip abroad for holidays in the recent 6 months and visited Croatia, Italy, Spain, Portugal, Russia, Turkey and Germany. One donor had eaten some game, but no donor claimed that he/she was eating raw meat. On the basis of these results there is no reference to the source of the infection. The questions asked are only related to gastrointestinal problems, the occupational contact with animals and the eating habit. For further investigation of the possible source of the infection a more detailed questionnaire has to be prepared for the time of donation and for the follow-up testing.

### Sequence alignment and pyhlogenetic analysis of the HEV RNA ORF2 region

**Fig 1:**
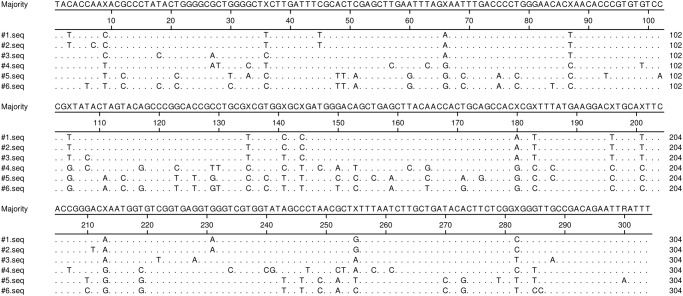
Sequence alignment. Comparison of a partial genome sequence in ORF2 from HEV positive samples. Dots indicate identity to the consensus sequence.

**Fig 2:**
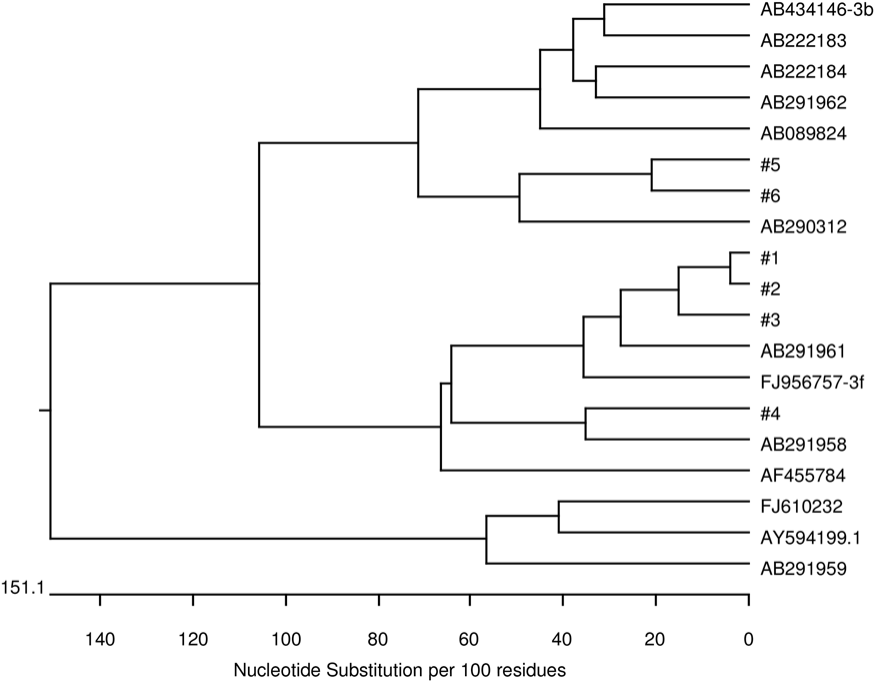
Phylogenetic relationship between genotype 3 HEV strains derived from Austrian blood donors (#1–6) and HEV reference strains (GenBank sequences are cited by their respective accession numbers). The tree was constructed by the Clustal W method on the basis of a 304-nt sequence fragment of ORF2 region.

All HEV RNA positive donor samples were genotyped and revealed genotype 3 isolates. To determine the relatedness between HEV isolates, a nucleotide alignment was performed (Fig. 1). Sequence homologies were between 80.9 and 99.3%. Sequences #1, #2 and #3 show the highest concordance with genotype 3f with nucleic acid identities ranging from 95.1–99.3% homology. A phylogenetic analysis of these sequences is illustrated in Fig. 2. Several attempts were made to obtain the sequence of ORF2 region of sample #7 with no success. Therefore, #7 was identified in the ORF1 region as genotype 3 (sequence and phylogenetic data not shown).

### Seroprevalence of anti-HEV IgG in Upper Austria

**Fig 3:**
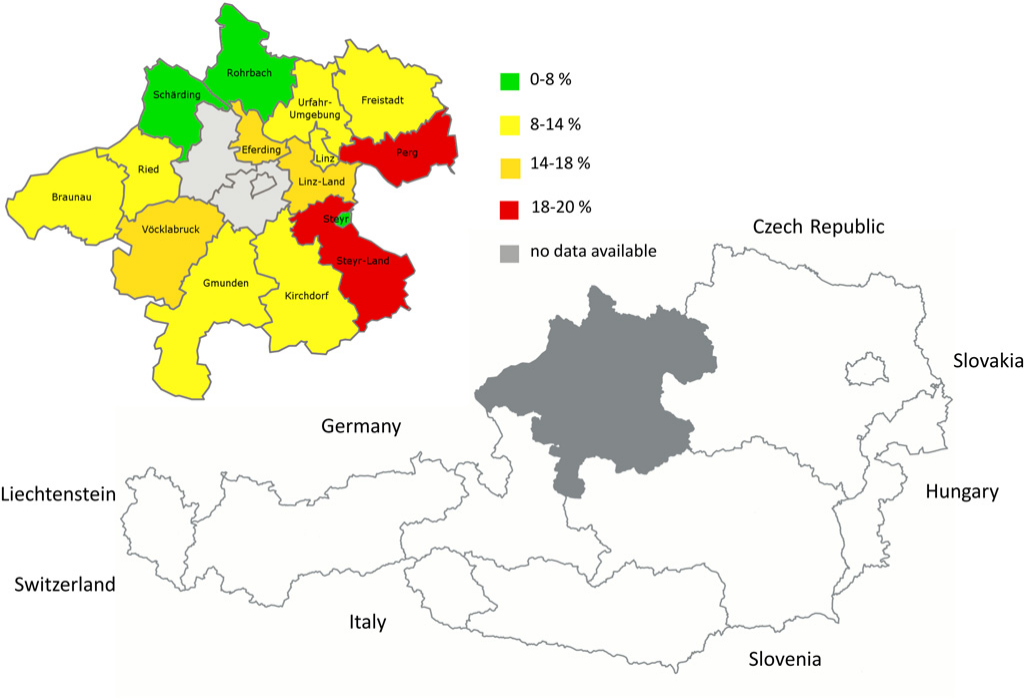
Geographical map of Upper Austria. A detailed analysis of seroprevalences [%] marked in green, yellow, orange and red of all districts is shown. Areas in grey are those with no available data. Copyright: The map of Austria is adapted from Statistik Austria and modified by Niklas N., http://www.statistik.at/web_de/services/interaktive_karten; The map of Upper Austria is adapted from wikipedia, author AleXXw and modified by Hofmann M., http://de.wikipedia.org/wiki/Datei:Karte_A_Ooe_ohne.svg

In total, 163 samples of 1,203 donations were tested positive for anti-HEV IgG antibodies, which relates to a seroprevalence of 13.55% (95% CI 11.6–15.5). Blood donors were set in relation to their place of residence in Upper Austria. The distribution of anti-HEV IgG positive donors over the districts of Upper Austria is shown in Table 2 and Fig. 3. There are no results for the districts of Wels, Wels-Land and Grieskirchen, as these districts are not tested by the local blood bank.

**Table 2:** Comparison of HEV seroprevalence in all districts of Upper Austria.

District	Number of donors	Anti-HEV IgG positive donors	% of anti-HEV IgG positive donors
Braunau am Inn	50	6	12.00%
Eferding	47	7	14.89%
Freistadt	121	15	12.40%
Gmunden	43	6	13.95%
Kirchdorf	36	5	13.89%
Linz	107	13	12.15%
Linz-Land	97	16	16.49%
Perg	138	29	21.01%
Ried im Innkreis	103	13	12.62%
Rohrbach	138	9	6.52%
Schärding	68	5	7.35%
Steyr	10	0	0.00%
Steyr-Land	65	12	18.46%
Urfahr-Umgebung	95	13	13.68%
Vöcklabruck	85	14	16.47%
**∑**	**1203**	**163**	

Regarding these results, there is a higher seroprevalence in the east (Perg, Steyr-Land; mean 20.01%) than in the north-western (Schärding, Rohrbach; mean 6.80%) part of Upper Austria (p < 0.05). The mean seroprevalence of all districts is 12.79%, within a range from 0% to 21.01% and a standard deviation of ±5.13. Steyr shows a seroprevalence of 0.00%, whereas this result might be misleading due to the low amount of only 10 participating donors.

**Fig 4:**
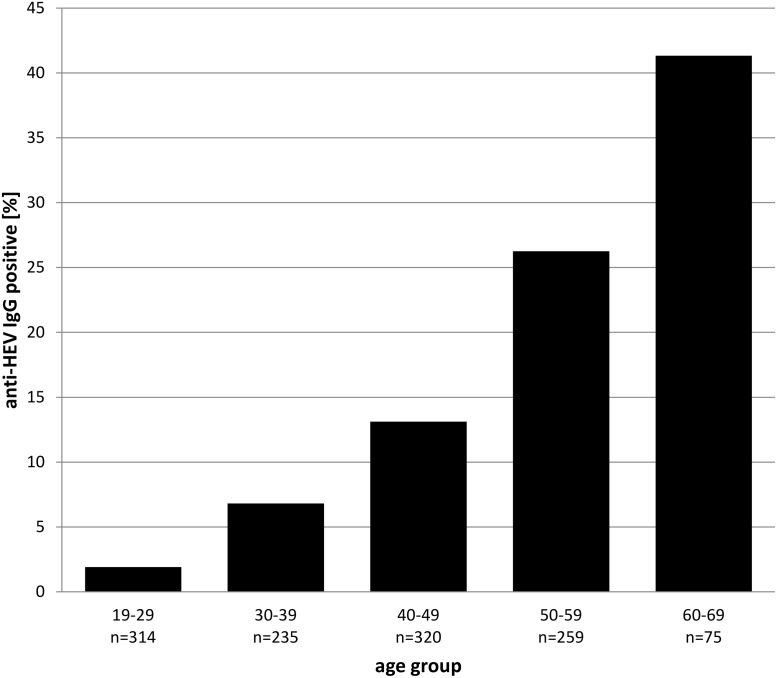
Prevalence of anti-HEV IgG. Estimated prevalence of anti-HEV IgG in 1,203 of Upper Austrian blood donors by age group.

The mean age of the tested blood donors is 40.49 years, within a range of 19 to 69 and the median age is 42. Mean age of the donors tested positive for anti-HEV IgG is 50.14, range 22 to 69 years. Categorized age groups revealed an age-dependent increase of HEV infection (r = 0.81; p < 0.01) (Fig. 4). Only 1.91% (95% CI 0.7–4.1) of the blood donors show positive results for anti-HEV IgG at the age of 19 to 29, whereas there are 41.33% (95% CI 30.1–53.3) positive for anti-HEV IgG at the age of 60 to 69 (chi-square p < 0.01).

These results show that there is a 20 fold increase of the risk to undergo a HEV infection at an advanced age (> 50) than at an early age (OR = 21.62). There is no trend of different distributions between male and female donors. 13.85% male and 13.08% female blood donors were positive for anti-HEV IgG (p > 0.1). Detailed results are supported in S3 Table.

## Discussion

The seroprevalence of anti-HEV IgG in the period of November 2013 to January 2014 amounts to 13.55% and our data reflects a cumulative increase with age. This confers to the wide range of seroprevalences in other European countries [25–27,29,30]. Lagler et al published the first large seroprevalence study of Austria civilians and military professionals and reported a seroprevalence of 14.3% with a very low representation of women (1.7%). Our findings are in line with this published data and we could confirm this seroprevalence rate in men (13.58%; n = 101) and women (13.08%; n = 62). However, differences in other countries may be influenced by different test systems with varying sensitivities, effects of sample selection e.g. age, exposure to contaminated food sources (uncooked meat) and contact to certain animal species e.g. pigs, wild boar and deer [25]. Possible differences in provinces in Austria published by Lagler et al could be explained by the diversity of farming, especially by the density of pig farms [25]. Nearly 40% of Austrians’ pig herds are situated in Upper Austria, where the most pig husbandries are located around the centre of Upper Austria and south of the Danube [34,35]. This distribution is not exactly in concordance with the distribution map of the appearance of anti-HEV IgG antibodies in this study, where the east of Upper Austria shows the highest seroprevalence and the north-western the lowest. It is controversially discussed, that living in high pig density areas is associated with a higher risk to undergo a HEV infection, but there are many studies that confirm that there is a tendency to higher seroprevalence in these regions [36–38].

The prevalence of HEV RNA in 58,915 blood donors in the Upper Austrian population resulted in 7 HEV RNA positive blood donors (0.01%). A questionnaire on behavioural aspects followed by an interview and liver-specific blood tests (e.g. alanine aminotransferase) have shown no significant symptoms or clinical findings, therefore there is no surrogate marker to indicate a hepatitis E infection in blood donors. Serological testing revealed that no donor was positive for either anti-HEV IgM or anti-HEV IgG antibodies at time of donation. At a follow-up testing all donors showed negative results for HEV RNA and anti-HEV IgG were positive. With the exception of one donor, all of 7 positive HEV RNA donors were anti-HEV IgM positive at follow-up appointment. The donor who showed negative results for anti-HEV IgM was tested 11 weeks after the donation, when IgM was already negative. The combination of PCR and serology reveals that all of the HEV RNA positive donors were in the seronegative phase of infection, and therefore posed a transmission risk without testing for HEV RNA. The normal ALT and CRP levels of the HEV RNA positive donors and the absence of any symptoms and antibodies at time of donation, confirm that a HEV infection in developed countries is difficult to diagnose without molecular methods. The serological testing of all HEV RNA positive donors implies negative results, which means that the only way to detect an HEV infection at an early stage is to perform a HEV RNA PCR.

Our findings for HEV imply a relative risk to find a viremic donor as in 1:8,416 (0.01%) donors, which is in line with data from some European countries like England 1 in 7,000 [39] and Sweden with 1 in 7,986 [40] donations. Lower rates were found in Scotland with 1 in 14,520 donations [41]. A far higher rate was found in Germany with 1 in 1,240 donations positive for HEV RNA [32]. On the one hand, these results can be explained by the variations of the occurrence of HEV infections within different regions and countries. The occurrence of HEV RNA positive blood donations was lower from May to mid- September 2013, which could be explained by the lower donation frequencies in this period or by different eating habits during the summer months than during fall and winter. On the other hand increasing the sensitivity of the assay by lowering the pool size to less than 96 samples could lead to a higher rate of detected HEV infections in blood donations. The nucleic acid concentration of the samples in this study ranged from 2.2 x 10^3^ to 2.9 x 10^5^ IU/ml with a mean of 1.1 x 10^5^. This is why we regarded the 95% limit of detection (LOD) of 11.6 IU/ml (7.48–25.09), which results in a LOD for a single sample of 1,113.6 IU/ml, adequate to detect HEV viremia in pools of 96 donations.

Based on our HEV RNA screening data of 58,915 samples with HEV RNA positive attack rate of 0.01%, there is a high risk of HEV infection from a blood product which might be given to immune compromised patients, a fact that might have been neglected. To ensure that at least these patients receive HEV free blood products, mandatory HEV screening should be considered in blood centres serving many young or immune compromised patients. In two published cases HEV was transmitted by transfusion of platelets, red cells and even Intercept-treated fresh frozen plasma and verified by 100% sequence homology of the transfusion-related isolates [15,22,42]. Despite of a few confirmed and published cases of transfusion-transmitted HEV infections consequences for young or immune compromised patients or those in poor health should be considered.

## Supporting Information

S1 Table HEV positiv blood donors (XLSX)

S2 Table IgG and IgM antibodies from HEV RNA positive donors. (XLSX)

S3 Table Anti-HEV IgG of the HEV RNA negative tested blood donors (XLSX)
